# Supplementary material for: Conditional chemoconnectomics (cCCTomics) as a strategy for efficient and conditional targeting of chemical transmission
Source: eLife. 2024 Apr 30;12:RP91927. doi: 10.7554/eLife.91927 (PMC11060718; doi:10.7554/eLife.91927)
Supplement: Figure 3—source data 3. [file elife-91927-fig3-data3.zip › Figure 3 source data3 part 2.zip!/Figure 3-Source data 3-part2/1002-9-CRISPResso_Report_e53dd8m5.zip!/CRISPResso_on_1002_9/1c.Alignment_barplot.pdf]

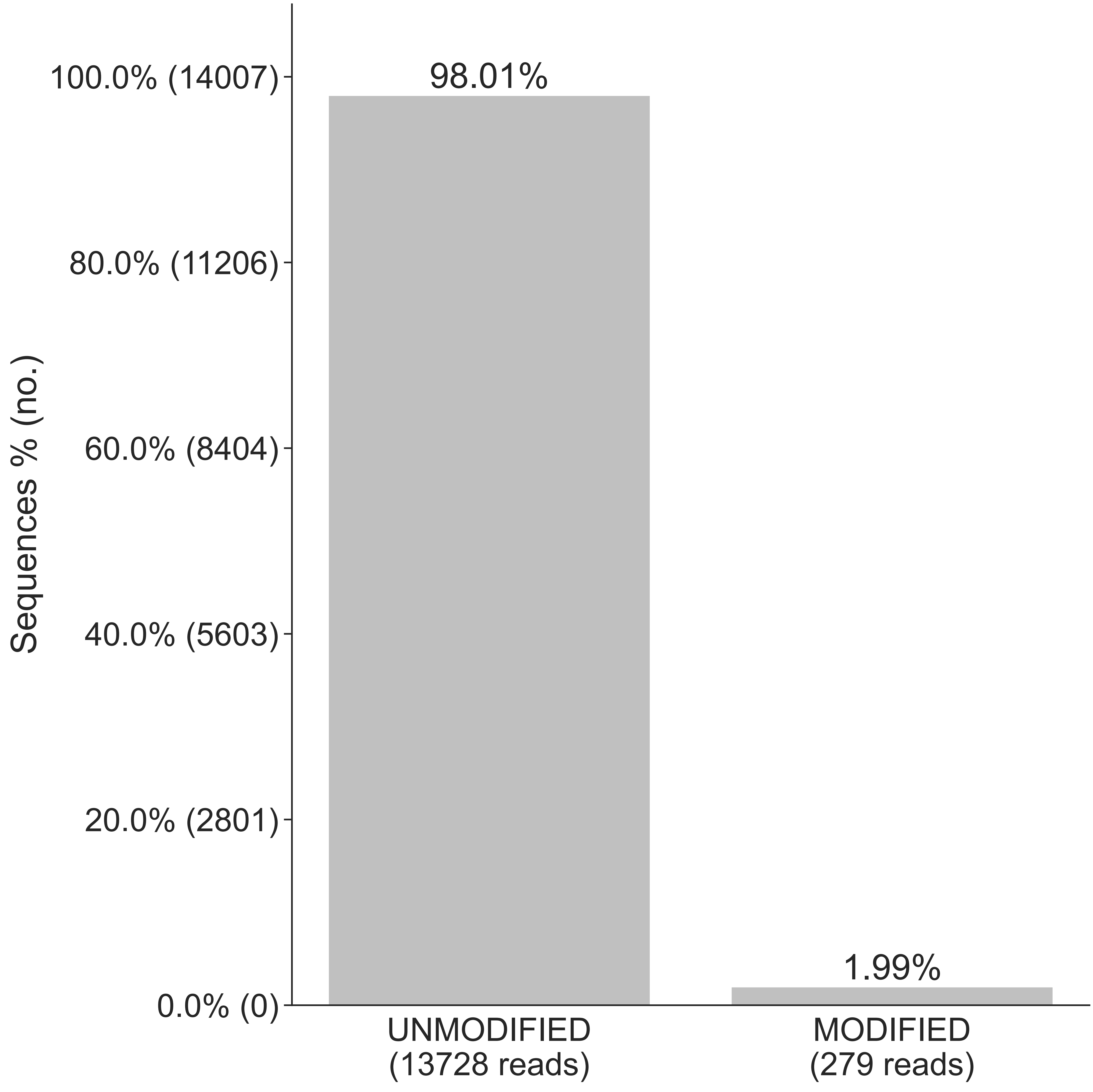

Sequences % (no.)
100.0% (14007)
80.0% (11206)
60.0% (8404)
40.0% (5603)
20.0% (2801)
0.0% (0)
98.01%
1.99%
UNMODIFIED
(13728 reads)
MODIFIED
(279 reads)
